# Prevalenceinstigating factors and help seeking behavior of physical domestic violence among married women of HyderabadSindh

**DOI:** 10.12669/pjms.301.4533

**Published:** 2014

**Authors:** Seema Bibi, Sanober Ashfaq, Farhana Shaikh, Pir Mohammad Ali Qureshi

**Affiliations:** 1 Dr. Seema Bibi, MBBS, FCPS, Department of Obstetrics & Gynaecology, Liaquat University of Medical and Health Sciences, Jamshoro, Sindh, Pakistan.; 2 Sanober Ashfaq, MBBS, DGO, MS, Department of Obstetrics & Gynaecology, Liaquat University Hospital, Hyderabad, Pakistan.; 3 Farhana Shaikh, MBBS, FCPS, Department of Obstetrics & Gynaecology, Liaquat University of Medical and Health Sciences, Jamshoro, Sindh, Pakistan.; 4 Pir Mohammad Ali Qureshi, MBBS, MPH, Department of Community Medicine & Public Health Sciences, Liaquat University of Medical and Health Sciences, Jamshoro, Sindh, Pakistan.

**Keywords:** Domestic violence, Risk factors, Help seeking behavior

## Abstract

***Background and*** ***Objectives*** ***:*** Domestic violence against women is highly prevalent but under reported issue having social, legal, health and economic implications. It needs to be identified and addressed in order to decrease the sufferings of women. Our objective was to find out prevalence, instigating factors and help seeking behavior of physical domestic violence against married women.

***Methods:*** A total of 378 married women who were attending Department of Obstetrics & Gynaecology, Liaquat University Hospital from January 1, 2013 to March 31, 2013 for different obstetrical & gynaecological problems were randomly selected and interviewed. After informed consent, required information was collected on predesigned performa including demographic details, prevalence, instigating factors, help seeking behavior for physical domestic violence.

***Results:*** About 31% (120) of women reported lifetime physical domestic violence. Husbands and in-laws were perpetrators in 70% (84) and 30% (36) cases respectively. Wives being disobedient and making arguments were the most common instigating factors for violence followed by husband’s drug addiction, extra marital relationship and infertility. It was severe enough to require medical care in 24% (29) cases. Only 2% (2) women sought social and legal aid.

***Conclusion:*** Domestic violence was quite common among married women, however help seeking was minimal. There is need to identify and address this menace effectively.

## INTRODUCTION

Domestic violence against women is common but underreported global epidemic having health, educational, legal, economic and above all human right implications. UNICEF defined the term domestic violence as violence against women and girls by an intimate partner, including cohabiting partner, and other family members, wherever this violence takes place and in whatever form.^1^ It is manifested as physical, sexual, psychological and economic abuse. Physical abuse includes slapping, beating, arm twisting, stabbing, strangling, kicking, burning, choking, threats with an object or weapon and murder. It is associated with adverse reproductive health consequences for women including unwanted pregnancy, miscarriage, pelvic inflammatory disease, sexually transmitted disease (STD), suicide, homicide and maternal mortality.

Worldwide women continue to suffer from this menace with World Health Organization (WHO) reported estimates of 10 to 69%.^2^ It is not uncommon in Pakistan. According to survey conducted by Human Rights Watch, about 70 to 90% women have suffered from some form of violence.^3^ Faryal Fikree and colleagues reported that 44% of women suffered from life time marital physical abuse in a cross sectional survey conducted in postnatal wards of tertiary referral hospitals of Karachi.^4^ In an another survey conducted in Rawalpindi and Islamabad, 97% of participants admitted that they had been subjected to some form of violence from verbal abuse to physical assaults or non consensual sex.^5^ It can be anticipated that domestic violence is prevalent but hidden reproductive health issue which needs to be recognized and addressed.

 This study was planned to find out the prevalence, instigating factors and help seeking behavior associated with physical domestic violence in married women in order to help health care providers and policy makers for designing and implementing preventive and treatment strategies for Pakistani women

## METHODS

A cross sectional survey was conducted in the Department of Obstetrics and Gynaecology, Liaquat University Hospital Hyderabad from January 1, 2013 to March 31, 2013. Approval was taken from Research Ethics Committee of Liaquat University of Medical and Health Sciences Jamshoro (Ref # LUMHS/REC/-106). All married women who were attending the hospital for different obstetrical or gynaecological problems as patients were included in the study. Unmarried Women and those who were not willing to participate were excluded. Verbal informed consent was taken before enrolling the participants. Simple random technique was utilized for enrolling study participants. Sample size of 378 subjects was used taking the frequency of physical violence as 44%^4^. They were interviewed face to face by principal investigator and her team members and required information was collected on predesigned performa. Data included demographic details, prevalence, instigating factors, help seeking behavior for domestic violence.

Physical domestic violence was defined as slapping, beating, kicking, arm twisting, hair pulling, stabbing, strangling and threats with an object or weapon by husband or any other family member.

***Statistical analysis:*** Data was entered and analyzed on SPSS version II. Simple frequencies and percentages were drawn. Instigating factors for physical violence were compared between physically abused versus never abused women. P value of <0.05 was considered significant.

## RESULTS

The prevalence of physical domestic violence was found to be 31% (120 out of 378 women). Husbands and in-laws were perpetrators in 70% (84) and 30% (36) cases respectively. Respondents were six years younger than their husbands with mean age of 29.87± 8.7 and 35.59 ± 11.98 years respectively. They have been married for 10 years (10.6 ± 8.38) and having four children (mean 3.59 ± 2.58). Average monthly income was about Rs.8000. Educational status of women was low in comparison to their husbands as 160(42.3%) women and 230 (60.8%) husbands were literate. Instigating factors for physical domestic violence are shown in Table-I. Physical violence was severe enough to require medical care in 24%(29) cases. Out of which 17%(n=21) attended family physicians clinic while 7%(n=8) required hospitalization.

Regarding help seeking behavior, only 2% (2) women sought social or legal help by reporting to police or NGO, while 50%(n=60) just complained to their parents and 48% (n=58) remained silent after sustaining physical violence.

## Discussion

Domestic violence is a social issue, hidden but prevalent in almost every society irrespective of race, region, religion, caste and language. It includes acts of physical, psychological and sexual abuse by someone who is involved in intimate partner relationship with the aim of controlling or punishing the victim. Most of the time husbands are perpetrators, but any member of the house hold can be involved. Our reported prevalence of 31% is in line with national figures quoted as 35% and 44% in various studies.^4^^,^^6^ Data from other developed and underdeveloped countries reflect similar situation. In an Indian study, 26% of women attending antenatal clinics reported physical domestic violence, mostly by their husbands.^7^ In another cross sectional survey conducted in emergency department of UK university hospital, revealed life time prevalence of domestic violence between 22.1% to 30.5% among women.^8^

**Table-I:** Instigating factors associated with violence between women abused/never abused

	*Risk Factors*	*History of violence(n=120)*	*No History of violence(n=258) *	*P. Value*
1	Wife Disobeying / Argues	70 (58.3%)	9 (3.5%)	0.00
2	Spouse drug addiction	52 (43.3%)	3 (1.2%)	0.00
3	Disliking by in-laws	29 (24.2%)	1 (0.4%)	0.00
4	Infertility	24 (20%)	4 (1.6%)	0.00
5	Spouse psychological problem	19 (15.8%)	0	0.00
6	Spouse extramarital relation	18 (15.0%)	0	0.00
7	Failure to produce male child	12 (10%)	2 (0.8%)	0.00

The most common instigating factors for physical domestic violence in our study was wife being disobedient or making argument with husband. Similar figures were quoted by Faryal Fikree and colleagues in a study done in three tertiary referral hospitals of Karachi.^4^ This highlights lack of women’s autonomy and decision making power related to her personal and family matters. Pakistani society is male dominant society, where women are supposed to be submissive and obedient for every decision, even pertaining to her household affairs, contraception, number of children and their education. Secondly they are illiterate, lack economic resources and supposed to obey their husbands according to socio-cultural norms. Jejeebhoy SJ and Sather ZA in their study conducted in India and Pakistan found restricted women’s autonomy in terms of decision making, economic empowerment, mobility and freedom. Moreover Pakistani women have less control over their lives as compared to their Indian counterparts.^9^ Therefore any deviation from society’s set norms, subject them to be disliked by their in-laws, as revealed by this study and ultimately being physically abused by whole family. Interestingly most of the instigating factors for violence were due to husband’s attitude and behavior including drug addiction, psychological disturbance and extramarital relations. Strong links of alcoholism and intimate partner’s violence have been reported from United Kingdom^8^, India^10^ and Uganda.^11^ Spouse extramarital relationship was another important risk factor noted in this study in line with international figures. In a nationally representative survey from Bangladesh, it was documented that wife abuse is associated with husband’s premarital and extramarital sex relations.^12^ Researchers have found that women who were exposed to intimate partner violence were at increased risk of sexually transmitted infections, including HIV.^13^^,^^14^ This may have roots with highlighted factor found in our study.

It is depressing to reveal that despite high level of domestic violence, rate of disclosure and help seeking behavior was minimal. Findings are consistent with reports from developed and under developed countries.^15^^,^^16^ Reasons for non disclosure are complex and multifactorial. Women continue to suffer in silence due to socio-cultural norms, misinterpretation of religious beliefs, subordinate status, and economic dependence. Other important barriers are fear of abuser, concerns for children, social isolation and lack of knowledge and familiarity with legal systems such as police and judiciary.^17^

Domestic violence has been associated with adverse reproductive health outcome including maternal morbidity and mortality. A report on confidential enquiries on maternal deaths in United Kingdom showed that lack or poor antenatal care was higher in women who died due to direct causes of maternal deaths. It also highlighted other indicators of domestic violence related to antenatal care including poor obstetrical history, unexplained admissions, non compliance with treatment regimens, recurrent sexually transmitted infections and constant presence of partner during consultation.^18^ Therefore there is dire need that all health care professionals should be aware of presence of violence, its causes, possible treatments and preventive measures. One of the simple and cost effective measures for identification of problem is incorporating violence history into routine history taking by health care providers particularly primary care physicians and obstetricians and gynaecologists.

In conclusion, domestic violence is not uncommon in our society and responses of women were open and positive on this sensitive issue. It is the responsibility of state, society and health care providers to plan and implement both preventive and treatment strategies in order to help women sufferers morally, medically and legally.

***Strengths and limitations of study: ***The strength of the study is reporting about a hidden, sensitive and rarely reported issue from the women of all age group, belonging to both rural and urban population from busy tertiary referral hospital having wide catchment area. There are certain limitations related to study design and data collection tool. The results could not be generalized as study is hospital based and cross sectional design has its own limitations. Recall bias may be present in responses as events of longer duration were questioned. Importantly, research study should be well designed and comprehensive with more specific and tested survey tool in order to understand dynamics of violence in special context of Pakistani population and to get real and valid data. 

## Authors’ contribution:

Dr. Seema Bibi conceived the study design, interpreted the results, and drafted the article. 

Dr. Sanober Ashfaq helped in designing the study protocol and collection of data 

Dr. Farhana Shaikh revised the final draft critically. 

Dr. Mohammad Ali Pir helped in acquisition and interpretation of data and also participated in writing. 

## Notes

***Etrhical approval: ***This study is done with the approval of Research Ethics Committee of Liaquat University of Medical & Health Sciences Jamshoro (Ref # LUMHS/REC/-106).
